# Detection of Porcine Parvovirus 2 (Ungulate Tetraparvovirus 3) Specific Antibodies and Examination of the Serological Profile of an Infected Swine Herd

**DOI:** 10.1371/journal.pone.0151036

**Published:** 2016-03-14

**Authors:** Attila Cságola, Zoltán Zádori, István Mészáros, Tamás Tuboly

**Affiliations:** 1 Department of Microbiology and Infectious Diseases, Faculty of Veterinary Science, Szent István University, Budapest, Hungary; 2 Institute for Veterinary Medical Research, Centre for Agricultural Research, Hungarian Academy of Sciences, Budapest, Hungary; Wuhan Bioengineering Institute, CHINA

## Abstract

Porcine parvovirus 2 (PPV2) is a member of a recently discovered group of swine parvoviruses occurring worldwide. It is frequently detected in lung samples suggesting some pathological role of the virus in diseases. To study this possibility an indirect ELISA was developed to detect PPV2 specific antibodies and to examine the serological profile of an infected swine herd where 185 serum samples collected from different age groups including sows were analyzed. According to the results maternal antibody levels decreased until 14 days of age and PPV2 specific antibodies started to rise between 28 to 43 days of age when respiratory signs were also observed in the examined swine herd. At 57 days of age the clinical signs disappeared and a rapid increase of PPV2 specific antibody levels could be measured simultaneously, peaking at 57 days of age. The viraemic status of different age groups was determined by qPCR using serum samples. At least a low level of viraemia was measured in every age group, but higher copy number of PPV2 was only detected at 57 days of age and the level decreased in older age groups. The changes in virus load and antibody levels together with the onset and decrease of clinical signs suggested that PPV2 had a role in the development of respiratory signs.

## Introduction

The development of molecular biology based detection methods led to the discovery of several new viruses among them a new parvovirus of pigs, namely porcine parvovirus 2 (PPV2) [1]. Parvoviruses are small non-enveloped viruses with single-stranded, linear DNA genomes of approximately 5–6 kilobases (kb). According to the current taxonomic classification (International Committee on Taxonomy of Viruses, ICTV, http://ictvonline.org/virusTaxonomy.asp) *Parvoviridae* family is divided into two subfamilies: *Densovirinae* and *Parvovirinae*. Members of *Densovirinae* infect various arthropods, while viruses belonging to the *Parvovirinae* subfamily are detected in vertebrates and form eight distinct genera, including Tetraparvovirus.

PPV2 (proposed name Ungulate tetraparvovirus 3), a member of the Tetraparvovirus genus, was discovered in 2001 during a survey for hepatitis E virus (HEV) in swine sera collected in Myanmar [1]. The HEV-specific primers non-specifically amplified a DNA fragment, which later proved to be of parvovirus origin. The amplification of nearly complete genetic material of the virus resulted a 5 kb long genome (H-1 virus, Myanmar-type PPV2), showing the highest similarity to Muscovy duck parvovirus and Bovine parvovirus 3 [1]. The occurrence of PPV2 appeared to be unique until many years later a genetically highly similar virus (Cnvirus) was detected in China [2] in blood samples collected between 2006–2007 from pigs affected by porcine circovirus 2 (PCV2) associated disease (PCVAD) and "high fever" disease (caused by porcine reproductive and respiratory syndrome virus, PRRSV). Genetic comparison of H-1 (Myanmar) and Cnvirus revealed approximately 95% identity [3]. Further studies indicated that PPV2 was presumably present worldwide as it was detected not only in Asia but also in Europe [4,5] and North America [6–8]. The report by Opriessnig et al. [7] showed that PCVAD affected pigs were infected with PPV2 with high prevalence and suggested a role of the virus as co-factor also in the porcine respiratory disease complex. Attempts to propagate the virus *in vitro* failed so far therefore animal experiments to fulfill Koch's postulates and to study the pathogenicity of PPV2 are not yet possible. In order to assess the possible pathogenic role of PPV2 without the use of animal experiments it is necessary to develop diagnostic methods for the *in situ* detection of the virus in tissue environment and also to detect and monitor the antibody response to PPV2 in context with the appearance of clinical signs.

The aim of this study was to develop an ELISA method suitable for the detection of PPV2 specific antibodies. Using this method we measured the specific humoral immune response against PPV2 in an infected swine herd and compared the antibody levels with the presence of the virus in serum samples.

## Materials and Methods

### Ethics Statement

The animal experiments were carried out in accordance with the Guidelines for Animal Experiments of the Szent István University and with EU Directive 2010/63/EU. The protocol was approved by the Committee on the Ethics of Animal Experiments of the Szent István University and the Central Agricultural Office (Directorate of Animal Health and Animal Welfare, Budapest, Hungary, Permit Number: PEI/001/960-3/2013). The animals were carefully monitored for any sign of distress, and all efforts were made to minimize animal suffering. Rabbits did not show clinical signs throughout the study.

### Sample collection

Lung samples were collected from four different Hungarian swine herds to determine the presence of PPV2. The samples originated from the South-Pest County Agricultural co. Ltd. (2713 Csemő, Határ road 1. GPS: 47°06'31.4"N 19°42'14.6"E), Deka-Hyb Pork Producer and Distributor Ltd. (3388 Poroszló, 093/10., GPS: 47°39'53.32"; 20°36'43.01"), FÜZES-FARM-95. Ltd. (3390 *Füzesabony*- Pusztaszikszó *0308/5*., GPS: 47°44'45.5"N 20°24'49.9"E) and Kossuth 2006 co. Ltd. (5123 Jászárokszállás, Adácsi road GPS: 47°39'30"N 19°58'17"E). Sample collection from deceased or slaughtered animals was part of the regular survey for diagnostic purposes of viral and bacterial infections of pigs and as such no specific permissions were required.

The detection of viruses and the determination of nearly full length genome of PPV2 was carried out as described earlier [4,5]. Based on the results, one PPV2 (GenBank code: KP765690) positive swine herd with 1600 sows producing 35000 pigs a year, was selected and examined further. All of the production phase was on the same farm, following the all in all out system. The examined herd was a farrow to finish herd where weaning was practiced at 4 weeks after farrowing when the piglets were transferred to the nursery units. In nursery units, there were two rooms per barns and 42 batteries in each rooms. Ten pigs were placed in each battery. At 55 days of age, pigs were delivered to the fattening units and remained there until slaughter weight was reached. There were two rooms per barns in fattening units, too, where 550 pigs were placed in 28 pens in each rooms. The herd is infected with swine influenza virus (SIV) and PCV2, but free from PRRSV and Pseudorabies virus. The pigs are vaccinated against PCV2 (3 weeks of age) and *Mycoplasma hyopneumoniae* (4 weeks of age), the sows against porcine parvovirus 1 (PPV1, Ungulate protoparvovirus 1), PCV2, erysipelas and *Eserichia coli* enterotoxemia. According to the herd veterinarian coughing, sneezing, conjunctivitis, fever and decrease of appetite was observed at 2 to 3 weeks after weaning. These signs were typically observed in several consecutive cohorts on the farm over a 1.5 year period.

Altogether 185 serum samples from different age groups were collected in this herd, by supplying veterinarian. Eighty samples were collected (5 litters/age groups, 3 samples/litter) from 2, 7, 14 and 21 days old piglets (60 samples) and their sows (20 samples), and 15–15 samples from 28, 36, 43, 57, 90, 120 and 150 days old growing and finishing pigs (105 samples). The litters and animals were chosen randomly in each barn. The sampling protocol and the precise origin of samples in each age group are detailed in S1 Appendix and S1 Table.

Five serum samples used as negative control were derived from specific pathogen free swine, originating from the Directorate of Veterinary Medicinal Products of the National Food Chain Safety Office (NFCSO, 1107 Budapest, Szállás u. 8. GPS: 47°28'33.4"N 19°07'45.5"E).

The all serum samples were stored at -20°C until examinations.

### Quantitative PCR

For PPV2 copy number quantification the capsid protein coding ORF2 was amplified with PPV2NotI and PPV2PacI primers and cloned into the pBacPAC9 (Clontec) plasmid digested by *Pac*I and *Not*I (Thermo Scientific). The primer sequences used in this study are listed in Table 1.

**Table 1:** List of primers used in this study.

Primer name	Sequence (5’-3’)	Reference
PPV2NotI	GATTAGCGGCCGCCATGAGCGCTGCCGA	This study
PPV2PacI	CGGTTAATTAATTATACACGATGAGCGCGT	This study
PPV2AF	ACACGATGAGCGGTACGA	[4]
PPV2AR	TCCTCACGAGGTCTCTTCTG	[4]
PPV2-EcoRI-2035F	GGAATTCTTTCCCAGTCTCGAACC	This study
PPV2-XhoI-2826R	GGCTCGAGCACCTGCGGCTGCAT	This study

Enzyme cleavage sites are underlined.

The insert bearing plasmid was propagated in TOP10 cells (Invitrogen) and purified by EZ-10 Spin Column Plasmid DNA Minipreps Kit (Bio Basic), based on the user manual. The presence and sequence accuracy of the PPV2 fragment in the plasmid was confirmed by sequencing. The amount of purified DNA was determined by NAS-99 NuDrop Micro-volume Nucleic Acid spectrophotometer at 260 nm, and the copy number was calculated (URI Genomics & Sequencing Center, http://cels.uri.edu/gsc/cndna.html). To determine the copy number of PPV2 nucleic acid in serum samples, ten-fold serial dilutions were prepared from the plasmid and amplified as controls by qPCR parallel with the samples in an Eppendorf realplex2 Mastercycler ep gradient S thermal cycler instrument. The standard curve was generated by triplication of plasmid dilutions. The correlation coefficient and efficiency of the standard curve was calculated by the built-in Eppendorf software.

Total nucleic acid was extracted from all of the 185 serum samples with GeneJET Viral DNA and RNA Purification Kit (Thermo Scientific), according to the given protocol. PPV2 DNA was amplified with PPV2AF and PPV2AR primers (Table 1) [4], suitable for the detection of both the Myanmar-type and the Cnvirus-type sequences. The specificity of the diagnostic primers was tested and described earlier [4].

The reaction mixture contained 1μl of each primer (25pM), 12.5μl of Maxima SYBR Green qPCR Master Mix (Thermo Scientific), 2μl sample and distilled water to a final volume of 25μl. The nucleic acid amplifications were as follows: UDG pre-treatment for 2 min at 50°C, preheating for 10 min at 95°C followed by 40 cycles at 95°C for 15 s, 60°C for 30 s, 72°C 30 s. The amplifications were completed with melting curve analysis.

### PPV2 antigen preparation

The protein sequence of PPV2 was aligned with that of structurally characterized viruses, like human parvovirus B19, PPV1 and adeno-associated virus 2. Based on structural alignments [9–11] the potential GH loop (surface loop between the G and H β strands of the capsid protein) of the PPV2 capsid between amino acids 679 and 942 was selected for bacterial expression.

The 792 nucleotide long capsid protein (264 amino acids) coding sequences were amplified with the PPV2-EcoRI-2035F and PPV2-XhoI-2826R primers (Table 1) specific for both the Myanmar-type and Cnvirus-type viruses. The Myanmar-type virus originated from the chosen herd and the Cnvirus-type template was described earlier [4]. Both Myanmar-type and Cnvirus-type PCR products were cloned using *Eco*RI and *Xho*I endonucleases (Thermo Scientific) into the modified pBAD/Thio-TOPO vector (Invitrogen) [12]. The insert containing plasmids were propagated in TOP10 cells (Invitrogen), purified by EZ-10 Spin Column Plasmid DNA Minipreps Kit (Bio Basic) and confirmed by sequencing. The partial PPV2 capsid proteins were produced in BL21-CodonPlus (DE3)-RIL cells (Stratagene). Briefly, one colony of plasmid-containing cells was cultured overnight (37°C) in 5ml LB Medium (containing 100μg/ml ampicillin, Fluka). The overnight cultured cells were grown for one hour in 100 ml fresh, pre-warmed (37°C) LB Medium (containing 100μg/ml ampicillin) after which one ml of 20% L-Arabinose (Sigma-Aldrich) was added to induce gene expression. The induced cells were incubated for 4 hours, harvested by centrifugation and stored at -20°C until purification. Before and after the induction, 1–1 ml of each culture was taken and the protein production was monitored by SDS-polyacrylamide gel electrophoresis (SDS-PAGE).

The produced partial PPV2 capsid proteins were purified by Ni-NTA Agarose (Qiagene) under denaturing conditions (using guanidine lysis buffer), based on a published protocol (http://kirschner.med.harvard.edu/files/protocols/QIAGEN_QIAexpressionist_EN.pdf). The purified proteins were analyzed by SDS-PAGE. The amount of proteins was determined using the NAS-99 NuDrop Micro-volume Nucleic Acid spectrophotometer at 280 nm.

### Immunization of rabbits

The rabbits were purchased from the Olívia Ltd. (6050 Lajosmizse, Mizse Farm 94, GPS: 47°4'27.83"; 19°31'31.49") and housed individually in standard size, stainless steel rabbit cages at the experimental animal facility of the faculty, under natural lighting conditions. The animals *ad libitum* received commercial rabbit pellets, alfalfa hay and tap water.

Vaccination procedures were done according to the “Experimental animal ethics and rules” of the Szent István University, Faculty of Veterinary Science, Budapest, Hungary. Two 10 weeks old, out-bred female New Zealand White rabbits were immunized intramuscularly with 0.15 mg of purified Cnvirus-type protein, adjuvated with aluminum hydroxide. The immunization was repeated two-times with 3 weeks intervals. Two weeks after the last immunization, rabbits were euthanized and their blood was collected. The serum samples were tested by Western blot method.

### Euthanasia of rabbits

Rabbits were euthanized by intramuscular administration of xylazine hydrochloride (2 mg/kg), acepromazine maleate (l mg/kg) and ketamine hydrochloride (15 mg/kg), as pre-anesthetic medication and after 10 minutes 2 ml of T-61 Euthanasia Solution was injected into the marginal ear vein.

### ELISA

The purified antigens were diluted to 5μg/ml in phosphate buffered saline (PBS) and ELISA plates (Nunc, Immuno Medisorp FH) were coated with 100μl/well of the diluted antigen for one hour at 37°C when plates were washed three times with PBS containing 0.05% Tween 20 (PBST) and blocked by 200μl of 1% bovine serum albumin and 0.05% Tween 20 diluted in PBS (BSAT) for two hours at 37°C. After washing three times, sera (1μl diluted in 100μl BSAT) were added and incubated for one hour at room temperature (RT). After subsequent wash with PBST, the plates were incubated with peroxidase-conjugated anti-swine IgG (whole molecule, Sigma-Aldrich, diluted 1:30.000 in BSAT) for one hour at RT. Following the three washing steps, chromogenic substrate (3,3′,5,5′-Tetramethylbenzidine, Sigma-Aldrich) was added and incubated in dark for 20 min. The enzymatic reaction was stopped by 50μl of 2N H_2_SO_4_ and the color reaction was detected in an ELISA reader at 450nm. The positive-negative threshold value was determined as OD 0.24 by duplication of the mean OD of the five negative control samples.

The inter-rater agreement (Cohen’s Kappa coefficient) was calculated based on the results of the ELISA [13]. The significance of the differences in the ELISA values measured on the two different antigens in an age group was analyzed with paired samples t-test. The normal distribution of the values was previously validated by Shapiro-Wilk test [14].

### Western-blot analysis

Non-induced and induced, empty and partial PPV2 capsid gene bearing pBAD/Thio-TOPO vector containing cell lysates (10μl) and purified partial PPV2 capsid proteins (1μg) were separated by SDS-PAGE, and the separated proteins were transferred to Nitrocellulose membrane (Kisker). The membranes were blocked with BSAT for one hour at 37°C and washed with PBST, then incubated with 10μl rabbit or porcine serum samples -diluted to 10ml in BSAT- for one hour at 37°C. The unbound antibodies were removed by washing with PBST and the membranes were incubated for one hour at 37°C with 10ml peroxidase-conjugated anti-swine and anti-rabbit IgG (whole molecule, Sigma-Aldrich), each diluted to 1:5.000 with BSAT. After being washed with PBST, the membranes were incubated with the chromogenic substrate (3,3′-Dimethylbenzidine, Sigma-Aldrich). After 15 minutes, the enzymatic reaction was stopped, removing the substrate by washing the membranes under tap water.

## Results

### Detection of viraemia

The PPV2 detected in the examined swine herd was of the Myanmar-type (GenBank code: KP765690). Melting curve analysis showed that the PCR products were single and specific. PCR products were not detected in negative controls. The primer specificity was confirmed by BLAST analysis, the amplification efficiency was 0.94, the correlation coefficient was 0.994, intercept value was 32.46 and the slope was -3.487 of the associated standard curve. The sensitivity of the qPCR was determined to be between 2.15x10^4^-2.15x10^3^ copies/ml genome equivalents. According to qPCR results viraemia was detected in every age group, mostly below 6.25x10^3^ copies/ml (Table 2).

**Table 2:** Detection of viraemia in different age groups.

Age groups (days)	Number of animals	PCR positivity	
		Number (and %) of positive animals	Number (and %) of stronger positive animals
sows	20	9 (40)	0 (0)
2	15	15 (100)	0 (0)
7	15	11 (73.33)	0 (0)
14	15	9 (60)	0 (0)
21	15	7 (46.67)	0 (0)
28	15	10 (66.67)	0 (0)
36	15	1 (6.67)	0 (0)
43	15	1 (6.67)	0 (0)
57	15	11 (73.33)	6 (40)
90	15	8 (53.33)	3 (20)
120	15	4 (26.67)	1 (6.67)
150	15	8 (53.33)	0 (0)

Stronger positive criteria in this study: PPV2 genomic copies above 1x10^4^/ml.

Higher virus loads were measured in 57 days old pigs where 6 of the 15 serum samples were stronger PCR positive, containing PPV2 genomic copies of 4.20x10^4^/ml, 5.45x10^4^/ml, 9.48x10^4^/ml, 4.48x10^5^/ml, 5.06x10^5^/ml and 7.14x10^5^/ml. In the 90 days old age group 3 samples out of 15 resulted stronger PCR positivity, containing 2.19x10^4^/ml, 3.76x10^4^/ml and 1.16x10^5^/ml copies and among the 120 days old pigs only one sample was stronger PCR positive: 1.53x10^4^/ml PPV2 (Table 2). All of the stronger PCR positive samples carried PPV2 specific antibodies in ELISA.

### Examination of PPV2 protein produced in bacteria

**Fig 1:**
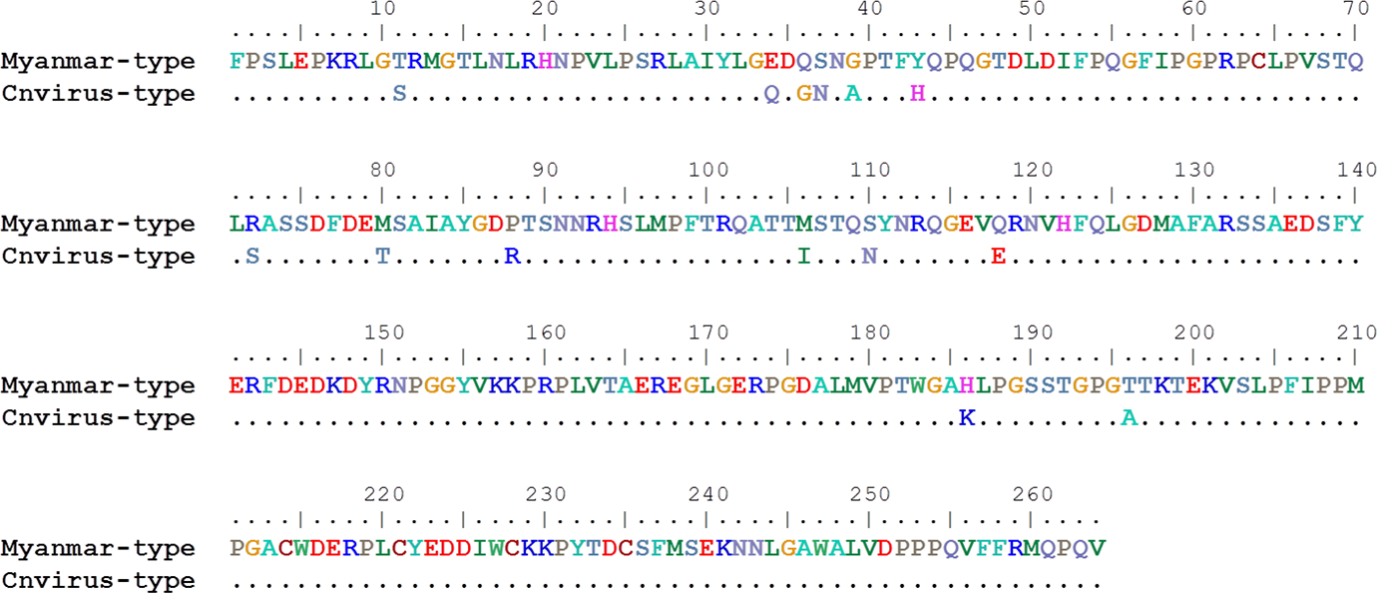
Comparison of the Myanmar-type and Cnvirus-type capsid protein amino acid sequences used in this study.

**Fig 2:**
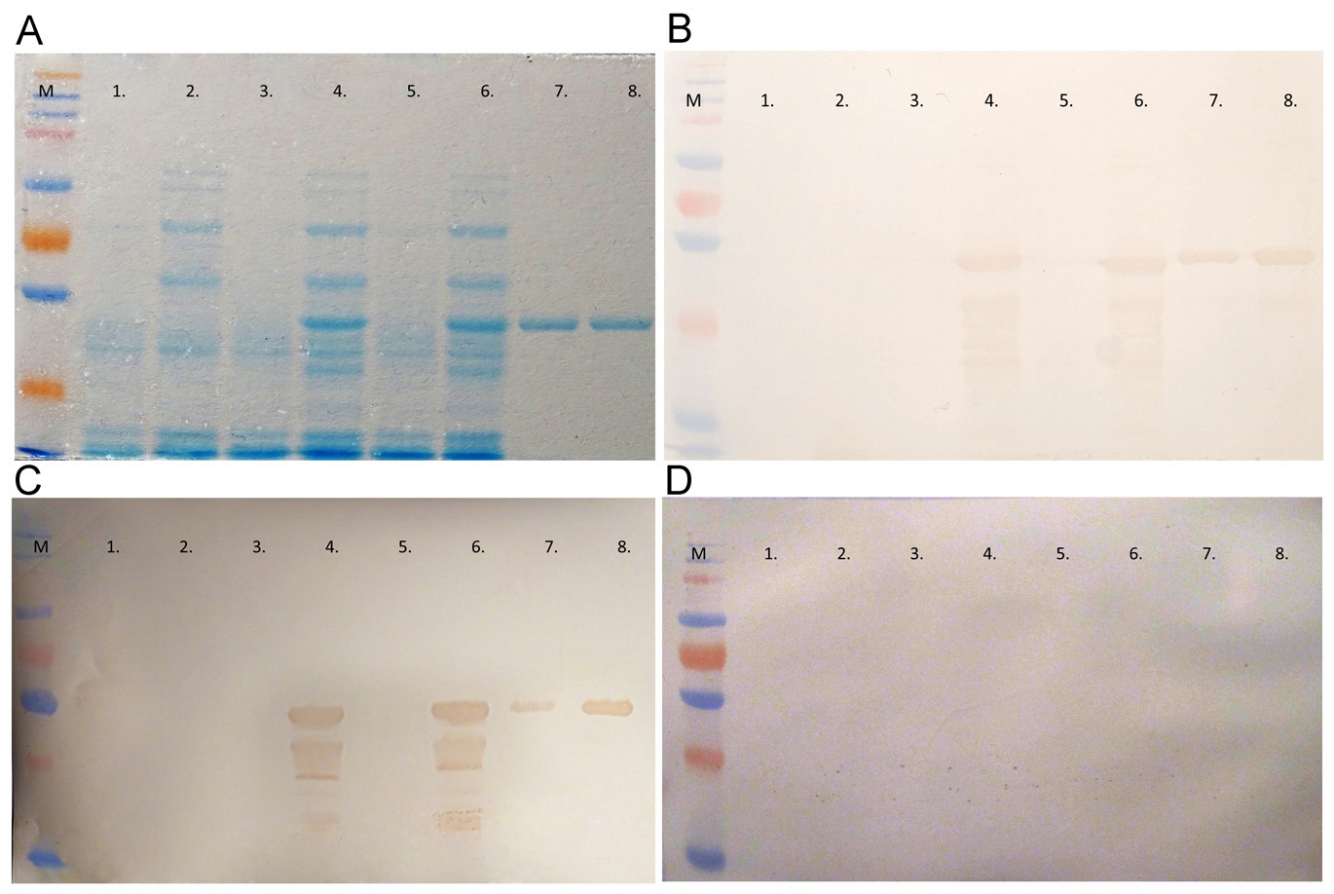
Analysis of non-induced, induced and purified PPV2 protein. (A) SDS-PAGE analysis. (B) Western-blot analysis with PPV2 positive rabbit serum. (C) Western-blot analysis with PPV2 positive pig serum. (D) Western-blot analysis using PPV2 negative pig serum. M: molecular weight marker (ProSiver QuadColor Protein Marker, Lonza); Lanes 1–6: cell lysates of bacteria with: 1. Non-induced, empty plasmid, 2. Induced, empty plasmid, 3. Non-induced Cnvirus-type PPV2 bearing plasmid, 4. Induced Cnvirus-type PPV2 bearing plasmid, 5. Non-induced, Myanmar-type PPV2 bearing plasmid, 6. Induced, Myanmar-type PPV2 bearing plasmid, 7. Purified Cnvirus-type antigen, 8. Purified Myanmar-type antigen.

The Myanmar-type and Cnvirus-type viruses differ from each other in 14 amino acid residues within the expressed region (Fig 1). The protein production of induced and non-induced bacterial cells was examined by SDS-PAGE (Fig 2A). Both types of PPV2 partial capsid proteins were produced in sufficiently high amount to allow purification. The purified proteins were also examined by SDS-PAGE (Fig 2A). The reactivity and purity of produced proteins were analyzed through Western-blotting. The SDS-PAGE separated cell lysates of induced and non-induced, empty plasmids and PPV2 genomic insert carrying plasmids containing bacteria, as well as purified proteins were transferred to Nitrocellulose membranes. The membranes were examined with sera of the immunized rabbits. None of the empty plasmid and non-induced plasmid carrying cells reacted with these sera, only the induced PPV2 partial capsid protein containing cell lysates and purified partial PPV2 proteins reacted (Fig 2B). Based on the results of ELISA, Western-blot analysis was performed with PPV2 positive and negative porcine serum samples. Similarly to rabbit sera only the induced PPV2 sequence containing cell lysates and purified PPV2 proteins reacted with pig sera that were positive in ELISA, and the negative serum did not show any reaction with these protein samples (Fig 2C and 2D).

### PPV2 specific antibody profile with Myanmar-type and Cnvirus-type antigens

**Fig 3:**
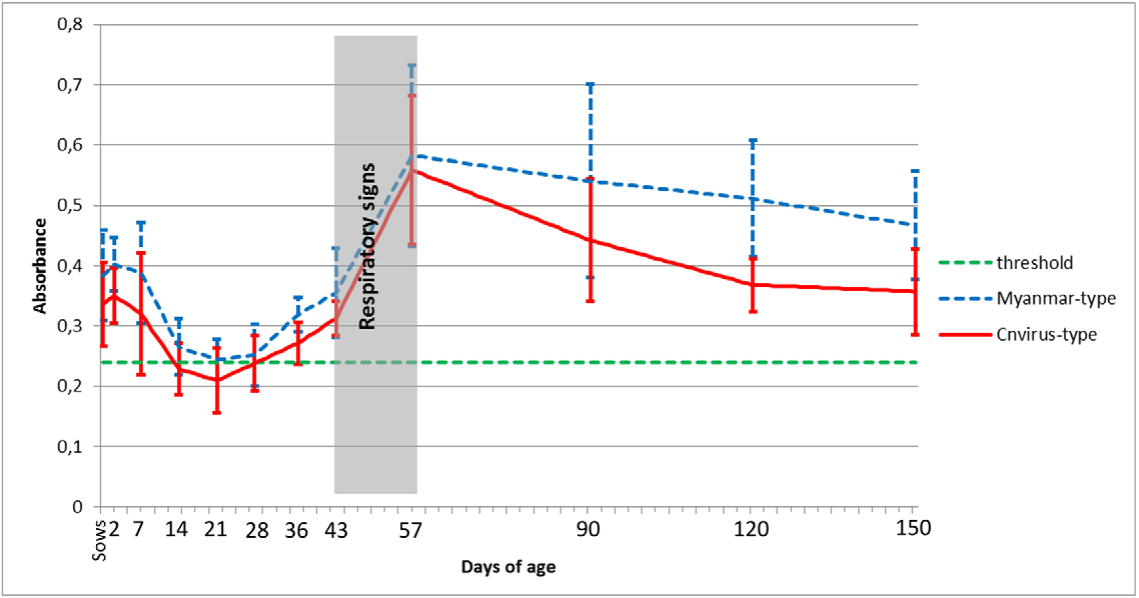
PPV2 specific antibody profile with Myanmar-type and Cnvirus-type antigens. The values show the average values of samples from animals of the same age, with standard deviations.

To determine the PPV2 specific antibody profile of an infected swine herd, serum samples were collected from different age groups (20 samples from sows, 15–15 samples from 2, 14, 36, 43, 57, 90, 120 and 150 days old pigs) and examined with the ELISA developed here. The results are shown in Fig 3. The serum samples of two days old piglets had higher antibody titers than their sow. The maternal antibody titer decreased until 14 days of age, PPV2 specific antibody levels started to rise between 28 to 43 days of age, and the peak was measured at 57 days of age. In order to detect immunogenic difference between the partial Myanmar-type and Cnvirus-type antigens, serum samples of the Myanmar-type PPV2 infected swine herd were examined with both types of the antigen. The antibody levels measured on the two antigens were very similar, but mildly stronger reaction was detected with the homologous Myanmar-type antigen at 36, 43 and 57 days of age, and clearly stronger reaction was measured at 90 days and over (Fig 3) when comparing results at group level.

The results of the ELISA’s were arranged in S1 Table and Table 3 and as it was expected moderate agreements were detected between the Myanmar- and Cnvirus-type antigen based ELISA tests by calculation of the kappa value (κ = 0.558) (Table 3). The number of the only Myanmar-type antigen positive samples (17 samples) was relatively higher than the number of the only Cnvirus-type antigen positive samples (3 samples) and this could influence the value of the kappa in a negative way. The most probable cause of the relatively high divergence in these two classes of samples is that the swine herd was infected by Myanmar-type PPV2.

**Table 3:** The agreement between qualitative test results obtained from ELISA based on two different antigens. Sera of the Myanmar-type PPV2 infected swine were investigated with Cnvirus and Myanmar-type antigens.

		Cnvirus-type antigen		
		+	-	
Myanmar-type antigen	+	149	17	166
	-	3	16	19
		152	33	185

Based on the paired samples t-test, statistically significant differences (p<0.05) were shown between the antibody levels on the two antigens at the 21 and the 43 days old piglets. Furthermore, more significant differences (p<0.001) were detected among the animals at the 2, 7, 14, and 90 days of age. Finally, the value of p was less than 0.0001 for the sow, the 36, 120 and 150 days of age.

## Discussion

Several new, emerging parvoviruses were discovered in pigs during the recent years [1,15–17] and according to the limited number of the reports these viruses are widespread in pig populations around the world. Clarification of the pathogenic role of the new parvoviruses including PPV2 is still pending mainly due to the lack of reliable *in vitro* virus propagation techniques for experimental infections. Swine herds are frequently infected not only with PPV2 but simultaneously also with one or more other pathogens like PCV2, PRRSV, SIV, *Mycoplasma hyopneumoniae*, *Actinobacillus pleuropneumoniae*, *Pasteurella multocida* and other microorganisms that hinder or make very difficult to clarify the pathogenicity of PPV2. Fortunately *in situ* detection methods and serological techniques at least in part may be of help to determine the importance of a PPV2 infection. In this study, we developed a serological method to examine the specific humoral immune response against PPV2 and determined the PPV2 specific antibody profile of on affected swine herd. The potential GH loop of capsid proteins of both Myanmar-type and Cnvirus-type viruses were chosen because the GH loop forms a large part of the parvoviral capsid surface around the threefold axis. Several important functions are localized in this region in different parvoviruses including tissue tropism, pathogenicity determining amino acids and most importantly antibody binding sites [18]. According to our best knowledge, this is the first description of serological examination of this virus infection. Based on our results, the piglets suckling colostrum from their PPV2 positive sows and absorbing high amounts of specific antibodies have very good passive, maternal protection. On the second day of life PPV2 specific maternal antibody level is higher in piglets than in their corresponding mothers, but this protection decreases rapidly (Fig 3). The increase of PPV2 specific antibody level starts after 28–36 days of age and calculating with the usual 7 to 10 days required for the primary immune response of B lymphocytes the estimated time of infection is suggested to be earlier than or between 21–36 days of age. In the examined swine herd, respiratory signs were observed 2 to 3 weeks after weaning (around 42–49 days of age) and the animals recovered, by 57 days of age these clinical signs disappeared. Along with this, after 43 days of age a rapid rise of PPV2 specific antibody levels could be observed, resulting high amounts of antibodies in the 57 days old group. The serum samples were also tested for PCV2 and SIV specific antibodies (data not shown), but the rise of PCV2 specific antibodies was observed only after 120 days of age and increase of SIV specific antibodies was not detected in pigs before, at and over 57 days of age. The examined swine herd was free of PRRSV, and the bacteriological examinations for common respiratory pathogens were also negative. These data suggest that PPV2 may have played a role in the development of the respiratory signs around 40–50 days of age. This assumption is supported by the frequent detection of PPV2 in pigs exhibiting respiratory diseases. In a previous study in Hungary 10.5% of the examined lung samples were positive for PPV2 [4]. In a Japanese swine herd, all of the 69 examined tonsil samples, collected from unhealthy 8 to 900 days old pigs were PPV2 positive [19]. In the USA the highest levels of PPV2 nucleic acid were present in lung samples originating from nursery and grow-finish pigs with a history of respiratory disease [8], covering the age of pigs where the respiratory clinical signs and PPV2 specific antibody rise were detected in our study. Based on a retrospective study PPV2 was present with high prevalence in archived North American lung samples from 1998 not only in PCVAD affected (33.3–55.6%) but also in PCV2 negative pigs (20%) [7]. The detection rate of viraemia in samples collected between 2006 and 2013 in North America was 27.3–41.4% in PCV2 positive and 33.3% in PCV2 negative sera [7]. Previously in China, PPV2 was identified in 9.66% of serum samples of PCV2 and PRRSV caused disease affected pigs 3 weeks prior the disease onset [2]. Similarly in the USA the peak of PPV2 viraemia was detected at 15 weeks of age, 2 weeks before the development of PCVAD [6]. In our study, low level of viraemia was detectable in every age group, but the peak was measured in 57 days old pigs (Table 2), earlier than in previous studies. It is likely that the antibodies produced by this age in increasing quantity and quality started to eliminate PPV2 from the blood stream. After the initial IgM antibody production the immune response normally produces IgG antibodies of increasing affinity and larger quantity. The more specific antibodies produced in later phase of the immune response showed stronger reaction with the homologous Myanmar-type antigen then the heterologous Cnvirus-type antigen as confirmed by the statistical analysis (Table 3) and shown by the ELISA results obtained for different age groups. This relatively small difference however did not influence the usability of the heterologous antigen in the serological diagnostic test.

Taking into account the few studies about PPV2 the results of this study suggest a pathological role of PPV2 in respiratory disease of pigs, but to clarify the importance of this virus further, well designed studies including experimental infections are needed.

## Conclusions

PPV2 is the most frequently detected emerging porcine parvovirus, usually present in the background of respiratory diseases. The rapid increase of PPV2 specific antibody level was measured in growing pigs parallel with the disappearance of respiratory signs. The highest PPV2 copy number was also detected in growing ages. The results and the few available studies on PPV2 suggest a possible pathological role of this virus in respiratory diseases of pigs.

## Supporting Information

S1 Appendix Sampling protocol. (DOC)

S1 Table Detailed ELISA results. (XLSX)
