# Supplementary material for: Detection of Porcine Parvovirus 2 (Ungulate Tetraparvovirus 3) Specific Antibodies and Examination of the Serological Profile of an Infected Swine Herd
Source: PLoS One. 2016 Mar 14;11(3):e0151036. doi: 10.1371/journal.pone.0151036 (PMC4790921; doi:10.1371/journal.pone.0151036)
Supplement: S1 Appendix — (DOC) [file pone.0151036.s001.doc]

Serum sample collection protocol

2 days old, 5 litter, 3-3 piglets/litter (15 sample) + sows (5 sample) of sampled piglets

7 days old, 5 litter, 3-3 piglets/litter (15 sample) + sows (5 sample) of sampled piglets

14 days old, 5 litter, 3-3 piglets/litter (15 sample) + sows (5 sample) of sampled piglets

21 days old, 5 litter, 3-3 piglets/litter (15 sample) + sows (5 sample) of sampled piglets

28 days old, 5 litter, 3-3 piglets/litter (15 sample)

36 days old, 5 litter, 3-3 piglets/litter (15 sample)

43 days old, 5 litter, 3-3 piglets/litter (15 sample)

57 days old, 5 litter, 3-3 piglets/litter (15 sample)

90 days old, 5 litter, 3-3 piglets/litter (15 sample)

120 days old, 5 litter, 3-3 piglets/litter (15 sample)

150 days old, 5 litter, 3-3 piglets/litter (15 sample)
